# Economic value of three grassland ecosystem services when managed at the regional and farm scale

**DOI:** 10.1038/s41598-022-08198-w

**Published:** 2022-03-09

**Authors:** Robert Huber, Solen Le’Clec’h, Nina Buchmann, Robert Finger

**Affiliations:** 1 ETH Zurich, Agricultural Economics and Policy, 8092 Zürich, Switzerland; 2 Department of Environmental Sciences, Environmental Systems, Analysis, Wageningen University, 6708PB Wageningen, Netherlands; 3 Department of Environmental Systems Sciences, Grassland Sciences, ETH Zurich, 8092 Zurich, Switzerland

**Keywords:** Environmental economics, Sustainability

## Abstract

Grasslands cover a major share of the world’s agricultural land and their management influences ecosystem services. Spatially targeted policy instruments can increase the provision of ecosystem services by exploiting how they respond to spatial differences in environmental characteristics such as altitude, slope, or soil quality. However, most policy instruments focus on individual farms, where spatial differences are small. Here we assess the economic value of three grassland ecosystem services (i.e., forage provision, carbon sequestration, and habitat maintenance) and its variability in a Swiss region of 791 km^2^ that consists of 19,000 farmland parcels when managed at the regional and farm scale, respectively. Our spatially explicit bio-economic simulation approach combines biophysical information on grassland ecosystem services and their economic values. We find that in our case study region, spatial targeting on a regional scale management increases the economic value of ecosystem services by 45% compared to targeting at farm scale. We also find that the heterogeneity of economic values coming from prices and willingness to pay estimates is higher than the economic gains from spatial targeting that make use of the spatial difference in environmental characteristics. This implies that heterogeneity in prices and/or societal demand of these three ecosystem services is more important for grassland management than spatial heterogeneity in our case study region. The here applied framework allows for an ex-ante assessment of economic gains from spatial targeting and thus provides basic information for the implementation of incentive mechanisms addressing the nexus of food production and ecosystem service provision in grasslands.

## Main

Grasslands cover a major share of the world’s agricultural area. They play a crucial role in global food security and grassland ecosystems contribute to human well-being trough the provision of a wide range of ecosystem services such as carbon sequestration, pollination or habitat maintenance^1,2^. Thus, grassland management has a major leverage effect on the sustainability of global agriculture. However, many trade-offs in the provision of grassland ecosystem services exist such as between forage provision and habitat maintenance^3,4^. Spatially targeted policy instruments that incentivize how farmers manage their grasslands accounting for environmental characteristics on a regional or landscape scale is hypothesized to jointly increase the provision of ecosystem services^5–9^. Yet, most policy incentives to support ecosystem services are still addressing farmers’ individual management^10–16^. Focusing the policy target on landscape scale management and the coordination of farmers management provides a viable pathway for maintaining agricultural intensities while at the same time preserving ecosystem services and biodiversity^17–27^.

To tap into the full potential of landscape scale management of grassland ecosystem services, two important challenges must be addressed. First, economic gains of landscape scale management should include the societal demand for non-marketable goods and services^28,29^. Second, the evaluation of landscape scale approaches should also consider farm-individual constraints e.g., with respect to forage availability and political regulations^30–32^. A spatial mismatch emerges if provisioning services from grasslands with a private market value e.g., via dairy or meat production, are preferred over ecosystem services with public values e.g., the willingness to pay for cultural ecosystem services^33–35^. This might make spatially targeted policy incentives aiming to increase the provision of grassland ecosystem services on a landscape level inefficient^36^. For example, farmers may shift environmental-friendly activities to a point in space where they do not reduce forage production but also do not support the provision of other ecosystem services.

We here address the trade-offs in economic value from marketable and non-marketable ecosystem services and the cost-inefficiencies that arise from political incentives that aim to support grassland conservation on regional or landscape scales^37,38^. We provide a bio-economic modelling approach to assess potential economic gains from spatial targeting of land-uses on regional or landscape scales that explicitly considers farm-individual constraints. More specifically, we include farm-individual production constraints in our bio-economic assessment of three grassland ecosystem services on a regional scale accounting for private and public services from grasslands. We use the categorization of ecosystem services according to the CICES (Common International Classification of Ecosystem Services)^39^ and focus on forage provision, which is the basis for ecosystem services related to livestock production, carbon sequestration as an indicator of climate regulation services and the maintenance of habitats necessary for sustaining populations of species. Our model is parametrized based on two meta-studies on grassland ecosystem provision and valuation. We go beyond existing studies that consider opportunity costs in the calculation of trade-offs by explicitly including biophysical and willingness to pay estimates for different grassland types in our valuation. We address two research questions. First, how do space and variable prices affect the economic value of forage provision, carbon sequestration, and habitat maintenance in a multifunctional grassland region? Second, how does the scale of the targeted policy area, i.e., from single farms to the regional scale, affect the cost-efficiency of spatially targeted policy interventions?

To answer these questions, we combine results of two meta-regression analyses on grasslands that provide empirical information about ecosystem services from different grassland types^40^ and their economic value^41^. Based on this information, we develop a three-step spatially explicit bio-economic simulation approach. First, we use farm and spatially explicit census data for 556 farms with animal husbandry and 19′000 farmland parcels in a Swiss case study region of 791 km^2^ to calculate ecosystems service provision (i.e., related to forage provision, carbon sequestration and habitat maintenance) from permanent grasslands in biophysical units. The scale of the case study region allows to include a gradient of various landscape archetypes from high intensity livestock farming to low intensity grassland areas. Second, we monetarize ecosystem service values on each parcel using forage and carbon price information for the valuation of forage provision and carbon sequestration respectively as well as willingness to pay estimates for maintaining extensively managed grassland. Third, we calculate the change in the value of grassland ecosystem services for different shares of extensive grassland habitats and two scales of targeted policy levels, i.e., either the farm or the regional scale.

This approach allows us to assess regional scale management of permanent grasslands along the trade-off frontier of private and public economic gains of three ecosystem services. Our exemplary case study builds a promising basis to scale our assessment to other grassland-dominated landscapes with high environmental gradients and that are confronted with a wide-spread trade-off between agricultural intensification and ecosystem service provision. Information about the spatial heterogeneity of economic values is highly relevant to assess different incentive mechanism to promote and preserve ecosystem services in agricultural landscapes. Those include conservation or ecosystem service procurement auctions^42,43^, payments for ecosystem services^44^, agglomeration bonus schemes^19,45^, collective action schemes^11^ or landscape labelling^46^.

## Inefficiencies from regional scale management

The level of ecosystem services provided by permanent grasslands such as forage provision, carbon sequestration and habitat maintenance depend on both management (e.g., land use intensity) and environmental drivers (e.g., altitude, soil), so that the ecosystem service provision is highly site- and management-specific. Grassland management regimes of different management intensities, i.e., different grassland types, support different ecosystem services^40^. More extensively managed grasslands (e.g. with lower fertilization rates and fewer cuts) provide lower forage yields but can also provide higher potentials for carbon sequestration and are key habitats for species conservation^40^. Thus, the more extensively the grassland is managed (as a form of semi-natural habitat), the higher the non-marketable ecosystem services, and thus the ecological sustainability and ecosystem resilience^27^. In turn, however, the higher the share of extensively managed grassland, the lower total forage provision, i.e., a marketable ecosystem service. This negative correlation between ecosystem services leads to a trade-off in the economic value of intensively and extensively managed permanent grassland^3,38,47^.

**Figure 1:**
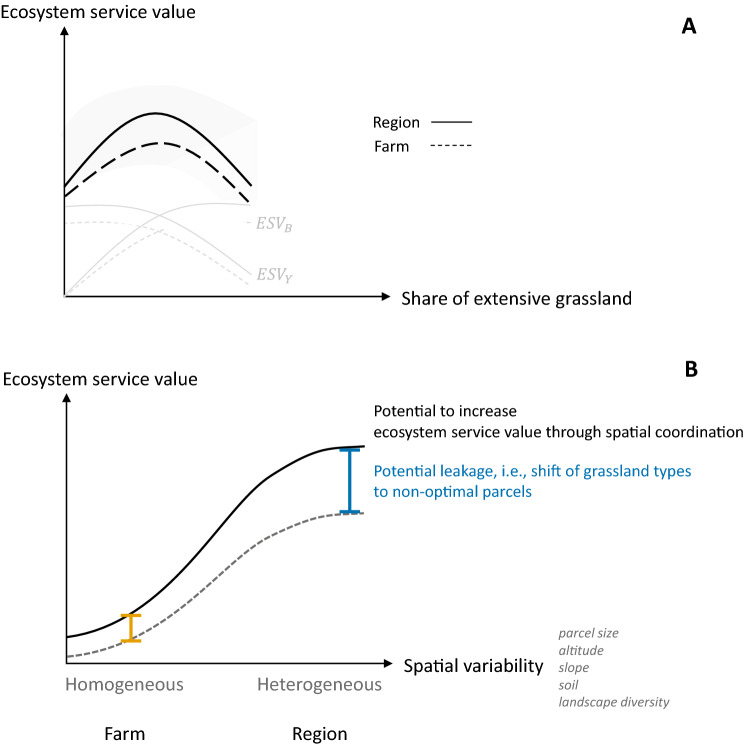
Conceptual synergies and trade-off in grassland ecosystem service values with increasing spatial extent. Panel **A**: Ecosystem service value (black lines) consists of marketable ecosystem service values ${\text{ESV}}_{{\text{Y}}}$ and non-marketable ecosystem services values ${\text{ESV}}_{{\text{B}}}$, which are here negatively correlated. With increasing shares of extensive grassland, there is a turning point at which the loss in value through reduced forage provision equals the value from non-marketable ecosystem services. Beyond this point, increasing the share of extensively used grassland reduces the overall value of permanent grasslands. With a regional scale approach (continuous lines), more ecosystem services can be provided compared to the farm scale (dotted lines). The light grey area underlying the ecosystem service value illustrates the conceptual uncertainty from aggregating different services. Panel **B**: Shifting the targeted level of policy incentives from the farm (yellow line) to the regional scale (blue line) increases ecosystem service values but also the variability of the services provided and thus the risk of inefficient policy interventions by shifting grassland management to non-optimal parcels.

With increasing shares of extensively managed grassland, we hypothesize that the economic value of grasslands increases as long as the additional economic value of non-marketable ecosystem service is higher than the reduction in the economic value of private marketable ecosystem services^14^. This is reflected in the hump shape relationship between the share of extensively managed grassland and the value of ecosystem services provided, i.e., the market values of forage provision, and carbon sequestration as well as the willingness to pay for cultural ecosystem services (cf. Fig. 1A). While other relationship may exist, a recent meta-study shows that most of the existing studies find a positive or strictly concave relationship between biodiversity and economic values^48^.

As spatial differences in environmental characteristics such as altitude, slope, soil quality or landscape diversity, i.e., the heterogeneity of land cover classes surrounding permanent grasslands affect the magnitude of ecosystem services, the provision of services within each grassland type can be highly variable^49^. Shifting grassland types in space allows taking advantage of how the different ecosystem services respond to this spatial variability^3,7^. Therefore, the economic value of grassland types depends on the spatial location of the grasslands, affected by two underlying mechanisms that drive the realization of ecosystem services and the corresponding economic gains gains^3,38^.

First, the higher the spatial variability, i.e., the more parcels differ with respect to environmental characteristics, the higher the potential economic gains from spatial targeting^13,50^. If all parcels would have the same environmental characteristics, spatial allocation of differently managed grasslands would not affect ecosystem service provision^40^. The spatial variability generally increases when enlarging the spatial scale^51^, e.g., from the farm to the region, and consequently also the overall economic gains from allocating grassland types differently in space (cf. Fig. 1B). Second, policy incentives that aim to optimize the provision of ecosystem services through the allocation of grassland management in space must consider the economic nature of the underlying values. With increasing spatial variability, the potential of an inefficient allocation increases because of farmers’ private optimization of marketable ecosystem services^13,52^. Combining these two mechanisms, we hypothesize that shifting the spatial scope from the farm to the regional scale increases the expected provision of multiple, but negatively correlated indicators of ecosystem services simultaneously increase potential cost inefficiencies from policy interventions. The form of the relationship between ecosystem service values and the inefficiency of the policy incentive will emerge from the spatial variability, the correlation between multiple services as well their economic values and remains an empirical and context-specific question.

We apply this concept in a Swiss case study by analyzing the potential economic gains and policy implications of a spatial redistribution of grassland management regimes and intensities using a spatially explicit bio-economic simulation approach. Our simulation approach accounts for variability across space and management and considers price and willingness to pay variability in the economic values of three ecosystem services. We here distinguish between marketable ecosystem services (forage yield in dt/ha and carbon sequestration in t/ha and year) and non-marketable values of habitat maintenance (number of vascular plant species per ha and year). To calculate the combined value of these ecosystem services, we use market prices for the economic valuation of forage provision and carbon sequestration as well as a benefit transfer approach of willingness to pay estimates for habitat maintenance.

The potential cost inefficiency is calculated by randomly distributing grassland types for two policy target scales, i.e., the farm or the regional scales. A random distribution of extensively managed grassland types in our approach implies that we refrain from an explicit simulation of the regulators’ and farmers’ complex decision making. Thus, our results represent the range of potential effects rather than a prediction of actual land-use. This can be used to inform policy making and instrument choice rather than a prediction of actual land-use.

## Results

**Figure 2:**
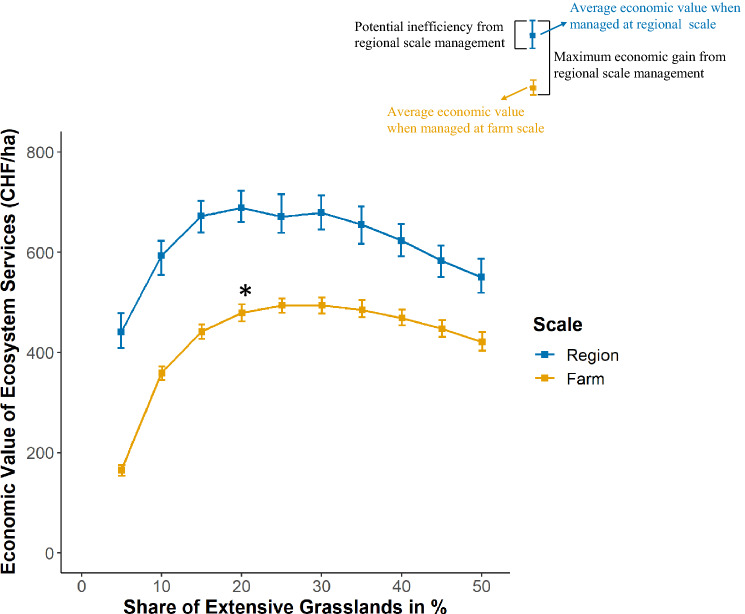
Economic value of three grassland ecosystem services with increasing shares of extensive grassland. Squares represent mean values compared to intensive land-use over 500 random allocations of four grassland types ${\text{x}}_{{\text{i}}}$ for two policy target levels ${\text{q}}$ (region, farm) with increasing shares of extensive grassland (0–50%). Error bars show minimum and maximum values reflecting spatial variability in ecosystem service values. Economic valuation is based on average forage price and mean willingness to pay estimates as well as a carbon price equivalent to the Swiss CO_2_ levy. The star (*) represents extent of extensive grassland use in the case study region in 2017.

Our analysis results in three main findings. First, in line with our conceptual framework, we find that the relationship between the shares of extensive grassland management and the economic value of forage, carbon sequestration, and habitat maintenance is not linear but follows a strictly concave form (Fig. 2). The additional economic value of three ecosystem services with increasing shares of extensive grassland ranged between 150 and 720 CHF per ha of permanent grassland. For lower shares of extensive grassland in the landscape, the economic value from increased plant species richness and carbon sequestration overcompensates for lower values of forage provision. With shares larger than 25% of all permanent grassland allocated to extensively managed grasslands, our simulation suggests that the private losses from reduced forage provision values exceed the economic values from the other two ecosystem services. This implies that additional shares of extensively managed grasslands do not further increase the economic value of the considered grassland ecosystem services. Thus, the consideration of non-marketable ecosystem service values increases the economic values of grassland ecosystem service only to a certain point.

Second, in line with the claim that landscape or regional scale management could alleviate the trade-offs between private and public ecosystem service provision^5–9^, shifting the policy target from the farm to the regional scale increases the average value of ecosystem service provision. This is represented by the gap between the average value of the here considered ecosystem services when managed at the regional and farm scale (cf. Fig. 2). In our case study, the value of the three ecosystem services increases on average by 45% (i.e., from 425 to 616 CHF per ha). Thereby, the difference between the value of grassland ecosystem services on the two spatial scales are higher with lower shares of extensive grassland. The higher the share of extensively managed grassland in the region, the closer the average economic value of grassland ecosystem services on farm and regional scale.

In addition, our results imply that the economic gains from regional scale management come with higher potential inefficiencies, i.e., the range of maximum and minimum values of the three ecosystem service values increases when shifting the policy target from the farm to the regional scale. This is represented by the larger error bars of the economic values on regional compared to the farm scale (a statistical summary of the results is presented in the supplementary material I). In our simulation, a shift of grassland types to locations where they effectively do not increase the non-marketable values of extensive grasslands, the economic gain of regional management is on average reduced by 34%. For example, a shift of the policy target level from the farm to the regional scale for a share of 25% of extensively managed grassland would have significant economic effects, i.e., increase the additional economic value by 208 CHF per ha. This increase would be achieved if grassland types are allocated to locations where they provide the highest ecosystem services. If these habitats are located where the underlying environmental characteristics result in lower ecosystem services, the potential economic gain from regional scale management is reduced by 78 CHF (the difference between the maximum and minimum of economic values on a regional scale). This implies that the extent of economic gains from shifting the policy target from the farm to the regional level critically depend on the spatial re-allocation of the extensively managed habitats to locations with high potentials for non-marketable ecosystem services. Thus, to tap into the full potential of regional scale management, coordination of the individual farm-level decisions would be indispensable^12,19^.

**Figure 3:**
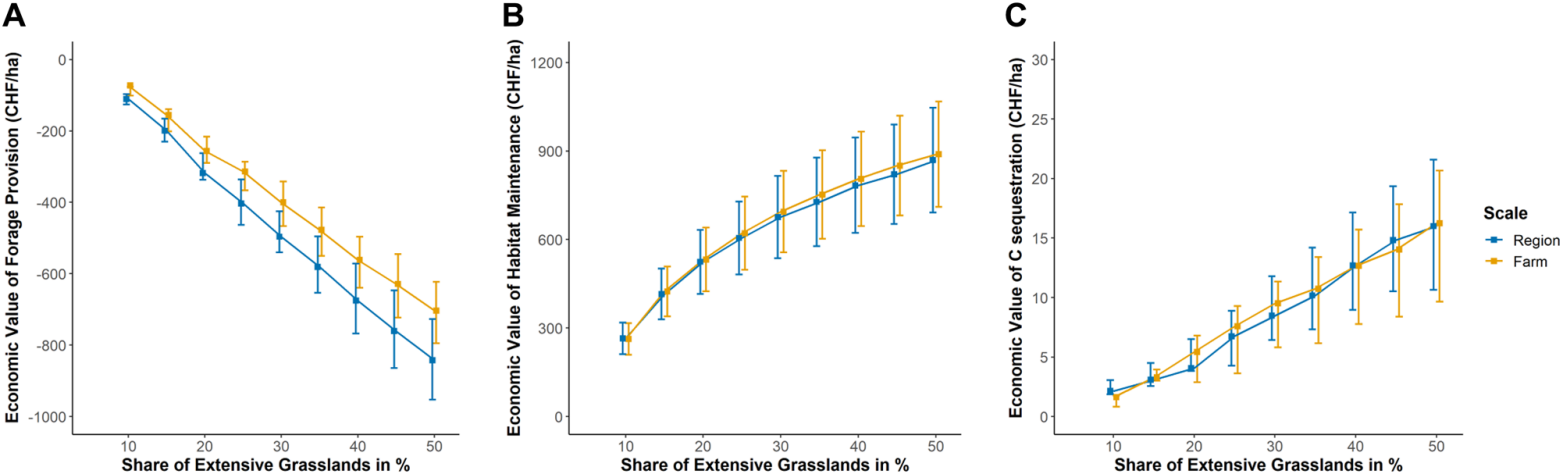
Economic value of forage provision, habitat maintenance and carbon sequestration from increasing the share of extensively managed grassland on farm and regional scale. Squares represent the change in mean values compared to intensive land use resulting from the random spatial allocation (500 simulation runs) for two spatial policy target levels ${\text{q}}$ (region, farm). Error bars reflect minimum and maximum values reflecting price and willingness to pay variability in ecosystem service values from variation in price assumptions: (**A**) Economic value of forage production with forage price variability. (**B**) Economic value of habitat maintenance using willingness to pay estimates. (**C**) Economic value of carbon sequestration with carbon price variability.

Third, our result showed that the variability of prices for forage and the uncertainty underlying the willingness to pay estimates were more relevant for potential inefficiencies than the variability of spatial environmental characteristics such as elevation and slopes^53^. Figure 3 presents the change in economic values with increasing shares of extensively managed grasslands for average forage provision, habitat maintenance and carbon sequestration when prices and willingness to pay estimates vary. Compared to a situation in which all permanent grassland is managed intensively, increasing the share of extensively managed grassland will reduce the contribution of forage provision to the economic value of ecosystem services (Fig. 3, Panel A). In contrast, the economic value of habitat maintenance and carbon sequestration increases with higher shares of extensively managed grasslands. The difference between minima and maxima of the economic value of forage provision and habitat maintenance coming from price and willingness to pay variability is 140 and 260 CHF per ha, respectively (average length of error bars in Fig. 3). This implies that the range of the economic value resulting from forage price variability and willingness to pay estimates is as high or higher than then the potential gain from shifting the policy target level from the farm to the regional scale. The economic gains from carbon sequestration in our case study are so small that a shift from the farm to the regional scale would not affect the overall economic value of the three ecosystem services much, irrespectively of the variability in carbon prices.

## Discussion and conclusion

We find economic gains from spatial targeting of extensively managed grassland in a multifunctional agricultural landscape. The economic benefit of regional scale management was higher if the share of extensive grassland was low and decreased with additional extensively managed grassland. This means that more non-marketable ecosystem services (i.e., in our case habitat maintenance) do increase grassland ecosystem service values only to a certain point^48^. However, the increase in economic value from regional scale management is smaller than the variability of the economic value from price and willingness to pay estimates. Thus, changes in prices or societal demand are more important for the economic value of grassland management than spatial heterogeneity in our case study region. From a policy perspective, this suggests that the identified heterogeneity in ecosystem service values might be much more important for the design of policy incentives than spatial heterogeneity^12,42,52^. For our case study region, this implies that schemes considering individual costs structures e.g., a procurement auction scheme might be more efficient than incentives for spatial targeting such as payments for ecosystem services^43,54^.

Our results also imply that there are considerable uncertainties from spatial targeting of ecosystem services on a regional scale. A non-optimal allocation of extensively managed grassland might lead to inefficiency, i.e., that policy incentives would not effectively increase carbon sequestration or conserve habitats. In our case study, such inefficiencies would reduce the economic gains from spatial targeting by at least 30%. This is at the lower bound of what existing studies identified when considering opportunity costs to calculate the economic value e.g., of bird diversity^13^. From a policy perspective, this finding implies that regional scale management needs some form of coordination between the individual farms to optimize the economic value of spatially targeted management. However, given the range of the variability in ecosystem service values from prices and willingness to pay estimates in our case study region, the economic gains from spatial targeting might not be enough to finance this coordination between the farms. Consequently, our case study, which represents a characteristic multifunctional landscape, implies that existing studies that do not consider farm-level constraints might overestimate the economic scope for improving grassland ecosystem service values through regional scale management. An additional aspect in this context is that many optimization approaches focus on ecosystem service indicators but do not jointly consider economic values of marketable and non-marketable goods and services.

The interpretation of our results come with certain restrictions. First, we do only consider three ecosystem services in our calculations. Extensive grassland might provide additional services such as pollination services or scenic beauty^2,40^. Adding more ecosystem services will increase the total value for landscapes with higher levels of extensive grassland and thus reduce the trade-offs between marketable and non-marketable grassland ecosystem services^48^ Thus, our results should be seen as a lower bound for the provision of non-marketable ecosystem services from grasslands. In addition, we are aware that forage yield, even though proposed as ecosystem service indicator in the CICES framework^39^ and applied in recent grassland ecosystem service assessments^55,56^, does not differentiate between the contribution by the grassland ecosystem and human inputs in the quantification of provision services^57,58^. Disentangling natures’ contribution in complex agri-environmental systems (here the interaction between grassland uses and livestock production) is an important avenue for future research^58–60^. In this context, our analysis does also not account for farmers’ reaction over time and in production, e.g. replacing grass with concentrate feed in feeding rations^38^ Adding external inputs like concentrate feed or chemical fertilizer could potentially reduce the ecosystems’ contribution to forage yields^58,61^. However, this would probably lead to negative environmental effects elsewhere and thus lead to leakage—i.e., the shift of environmental externalities in other countries such as soy production regions in the Brazilian amazon^37,38^. At the same time, farmers could also manage their grassland to increase the number of species within a given grassland type, which could positively affect forage provision and habitat maintenance^62,63^. To address such reactions the farmers’ complex decision with respect to grassland use under different incentive schemes would have to be explicitly simulated. Further research could specifically include farmers management decisions and how these affect natures’ contribution to a broader set of grassland ecosystem services^64^. Furthermore, the results may also be restricted to a certain size of the region and types of land-uses considered in the re-allocation. Shifting management across regions with different production patterns and ecosystems, e.g., also from permanent grassland to cropland or forest, might increase potential inefficiencies and create additional trade-offs between food production and ecosystem services. Finally, we are aware that using stated preference methods to monetarize non-marketable values of extensive grassland is not fully able to capture all dimensions of values that people might associate with extensive grassland^65^. However, we believe that the monetarization of grassland ecosystem services in our study provided an important basis to compare the influence of different policy target levels and at the same time considering spatial uncertainty as well as uncertainty of prices and the willingness to pay estimates.

Despite these restrictions, our results add a new perspective to the perspective of landscape or regional scale management of grassland ecosystem services. To be able to shift the targeted management level from the farm to the regional scale^8,20–26^, information not only about the spatial heterogeneity of ecosystem service provision is necessary, but also about farmers’ opportunity costs and the economic value of ecosystem services. Consider this information jointly allows to set à priori the type of policy incentive that is more effective in supporting grassland ecosystem services^12,19,44,50^. Our bio-economic modelling framework, integrating data on biophysical units of ecosystem services provides not only costs but also economic values for the assessment of different incentive mechanisms supporting ecosystem services. Transferring our approach to larger areas and more grassland ecosystem services could reconcile institutional as well as practical requirements e.g., when designing agri-environmental policies. This will allow to provide ex-ante information about incentive mechanisms supporting multiple ecosystem services, ready to cope with the challenges for agriculture in the future.

## Method

### Marketable and non-marketable value of ecosystem services

We here distinguish between marketable (forage provision and carbon sequestration) ecosystem services $ES_{y}$ and non-marketable ecosystem services (habitat maintenance) $ES_{B}$ using prices $p$ and willingness to pay estimates $WTP$ for their valuation, respectively. The basis for our calculation is the (counterfactual) situation in which all grassland parcels are managed intensively. This counterfactual allows to consider the trade-off between marketable and non-marketable along the gradient of intensively managed to very extensively managed landscapes. Following Fisher et al. (2008), we then calculate the additional i.e., the change in ecosystem service provision depending on the shares of each grassland type and their location. Thus, the value $ESV_{i}$ of each grassland parcel $i$ is:

1 $$ ESV_{i} = p*\Delta ES_{{Y_{i} }} + WTP*\Delta ES_{{B_{i} }} $$

with $\Delta ES_{{Y_{i} }}$ and $\Delta ES_{{B_{i} }}$ as the change in ecosystem service provision from each parcel relative to an intensively managed parcel. The ecosystem service value per ha of permanent grassland $ES$ that reflects this trade-off can then be calculated by the value of ecosystem services over all parcels with different environmental characteristic in the region (with $n = 19^{\prime}000$ parcels):

2 $$ ESV = \mathop \sum \limits_{i = 1}^{n} ESV_{i} $$

We here assume that the underlying environmental drivers related to a specific location of the grassland types is unobservable for the policy maker and thus represents a random variable in the calculation of ecosystem services. This uncertainty about the location of the grassland types results in spatial heterogeneity of the value of grassland ecosystem services. In addition, we assume farmers’ to be price takers, i.e., that changes in marketable ecosystem services do not influence their price. The prices, however, may vary between farms and over time. We reflect this price variability by randomly assigning prices to forage produced from different spatial allocation of grassland types based on the observed market prices in the past. Finally, we derive $WTP$ estimates using a benefit transfer approach based on a meta-study on the willingness to pay for extensively managed grasslands. We assume that our estimates are constant over time but decrease with higher provision levels^48^. The willingness to pay for non-marketable ecosystem services comes with high methodological uncertainty. We consider this uncertainty in the calculation of $ESV$ by considering maximum and minimum $WTP$ estimates from our benefit transfer approach in our calculations.

### Spatially explicit calculation of ecosystem services

We calculate forage provision, carbon sequestration and habitat maintenance from a region with $$n$$ farms cultivating $$i$$ parcels. To estimate the ecosystem service provision from different grassland types at different locations, we use results from the regression analysis by Le Clec'h, et al.^40^. We use forage yield ($$\widehat{{Y_{i} }}$$) as a proxy for forage provision, net carbon fluxes as indicator for carbon sequestration ($$\widehat{{C_{i} }}$$) and plant species richness ($$\widehat{{B_{i} }}$$) for a predictor of the suitability of the habitats to sustain populations of species. These proxy indicators are predicted for each parcel $$i$$, depending on grassland type $$x_{i} $$(extensive pasture, extensive meadow, intensive pasture, and intensive meadows) as well as the underlying production characteristics at a specific location, i.e., elevation, soil quality, slope, and landscape diversity. The prediction is based on various regression analysis using a broad set of data (census data, Corine Land Cover, Digital Elevation Model, FLUXNET2015 dataset, species richness measurements and yield data)^40^. Data analysis and documentation was done in R Studio 4.0.2 (see supplementary material II).

In addition to the location of the corresponding grassland type, the provision of grassland ecosystem services depends on the shares of the different grassland types $w_{x}$ within a region. To consider underlying production practices of the different farms, we assume that the relation between grassland and the total area for each farm remains constant and that farms do not switch their type of production. That implies that for a farm with a share of 60% of grassland, the sum of the different grassland shares $w_{x}$ also add up to 60% of the total area. Within the different production zones, i.e., sub-regions with the same agricultural production conditions, the relation between grasslands and total area is maintained. This assumption guarantees that the amount of forage yield estimated in our random assignment approximates the current production practices, and grassland does not replace crop production in our case study region.

#### Economic value of ecosystem services

To estimate the economic value of forage provision $(ESV_{Y} )$, we first multiply the predicted forage yield (in t per parcel) with the price for forage $p_{x}$ (in CHF per t) from extensively and intensively managed grassland respectively:

3 $$ ESV_{y} = \mathop \sum \limits_{i} \left( {\widehat{{Y_{i} }}*x_{i} } \right)*p_{x} $$

Please note that CHF are about equal in value to USD and Euros (in November 2021). We then calculate the difference between the initial economic value of forage yield with intensive grassland management only and the economic value with increasing shares of extensive grasslands $$ ESV_{Y} = ESV_{yMax} - ESV_{y}$$. This allows estimating the private opportunity costs a farmer has shifting from intensively to extensively managed grassland.

To calculate the value of carbon sequestration, we multiply net carbon flux (t C/ha/year), computed from net ecosystem exchange, carbon imports and exports through fertilization and harvesting, with the price of carbon $p$

4 $$ ESV_{C} = \mathop \sum \limits_{i} \widehat{{C_{i} }}*p $$

The economic value of habitat maintenance is calculated based on a meta-regression analysis of willingness to pay (WTP) estimates for ecosystem services from extensively managed grassland ^41^ The meta-analysis allows deriving the willingness to pay for extensive grassland management based on the size of the region, population size, income, and expenditure levels. In addition, the study allows differentiating between extensive grasslands in lowlands and mountain regions. Based on this meta-analysis, we use a benefit transfer approach to calculate the $WTP$ for habitat maintenance in our case study region^66^. Our benefit transfer approach has the following linear form:

5 $$ WTP_{i} = \hat{\beta }_{0} + \hat{\vartheta }_{i} y_{i} + \mathop \sum \limits_{g = 1}^{G} \hat{\mu }_{g} f_{g} + \mathop \sum \limits_{k = 1}^{K} \hat{\beta }_{k} z_{k} $$

where $WTP_{i}$ represents the willingness to pay estimate transferred from the meta-analysis to mountain and lowland fields in our case study region. The first term after the intercept $\hat{\beta }_{0}$ (i.e., $y_{i}$) is a dummy variable for whether the parcel is in the lowlands or mountains. The second term describes the case study characteristics where $f_{g}$ is a set of variables $g$ characterizing specificities of the case study (e.g., size of the region). The third term represents methodological characteristics in the meta-analysis where $z_{k}$ is a vector containing $k$ methodological variables of the valuation studies (e.g., dichotomous choice vs. multinomial choice setting). The parameters $\hat{\beta }_{0}$,$\hat{\vartheta }_{i} ,$ $\hat{\mu }_{g} , \hat{\beta }_{k}$ were generated from the meta-regression model by Huber and Finger^41^ using an MM-estimator, a robust regression technique^67^ and multi‐way clustering, which considers the adjustment of standard errors for variations of estimates from the same research article^68^.

We estimate the $WTP$ by adjusting the case study variables $f_{g}$ to Swiss specific parameters i.e., the size of the case study region, GDP in the year of the meta-analysis, real expenditure per capita in purchase power parity, general public, and the dummy variable for the region (mountain vs. lowlands). In contrast to case study related variables, there is no rule on how to consider methodological variables $z_{k}$ in the benefit transfer^69^. As a consequence, the calculation of willingness to pay estimates in a benefit transfer approach is associated with uncertainties from the underlying methods. Here we make use of this variability in the underlying stated preference studies to identify an uncertain range of $WTP$ in our simulations. Thus, the variability of economic values for maintaining habitats reflect the methodological uncertainty resulting from the underlying studies in the meta-analysis of willingness to pay for extensively managed grassland. Thereby, the largest methodological difference results between studies that apply a discrete choice experiment vs. studies that apply contingent valuations.

Since the studies used in the meta-regression did not report per ha willingness to pay, we assume that the predicted values ($\widehat{{WTP_{i} }}$) represent an upper bound of the total willingness to pay for our case study region with 50% of extensive grassland and the maximum number of species predicted. We use a log relationship between the share of extensive grasslands and its value. This relationship is then used to estimate the economic value of habitat maintenance for the share used in this study. This leads to a decreasing marginal benefit of habitat values in line with the foundations of economic valuation^70^ Finally, we calculate the overall economic value for habitats from different spatial grassland allocations in the case study region based on the spatial location of the corresponding parcel and the predicted number of species:

6 $$ ESV_{B} = \mathop \sum \limits_{i} x_{i} *\widehat{{WTP_{i} }}*b_{i} $$

with $b_{i} = \frac{{\widehat{{B_{i} }}}}{{\widehat{{Max(B_{i)} }}}}$ as a correction factor that adjusts the economic value of habitat maintenance from parcels in the lowlands vs. mountain regions to the predicted number of plant species in each simulation.

### Simulation of grassland allocation

**Figure 4:**
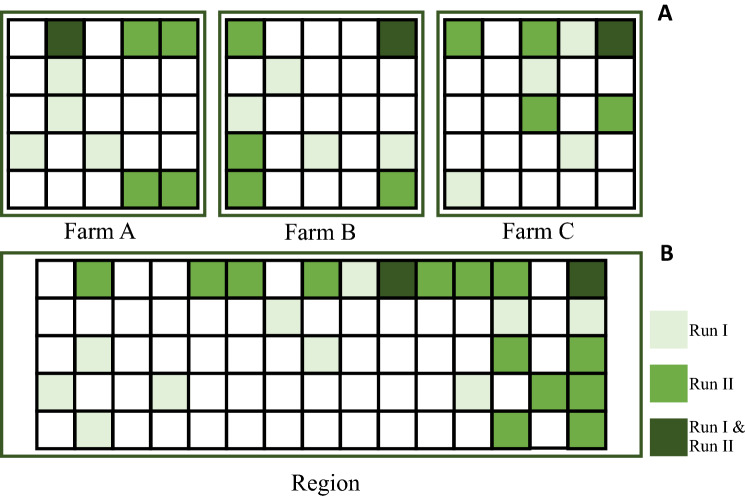
Schematic illustration of the random allocation of grassland types on different policy target levels. A given share of extensive grassland (here 20%) is randomly distributed (**A**) within each farm; (**B**) on regional scale. Green and light green cells represent two possible outputs of repeated runs. Dark green cells imply that the corresponding parcel had been randomly selected in both simulation runs. The white parcels represent all other agricultural land uses (intensive grassland and crop production). Please note that for illustrative purpose, parcels are represented as squares here. In our data, the effective size and shape of the parcels is considered.

We randomly distribute the grassland types $x$ on all available parcels $i$, so that the sum of all grassland areas equals the exogenously given shares $w_{x}$ (for data and simulation code in R see Supplementary material II). We apply this random distribution of $x_{i}$ to two policy target scales $q$, i.e., the shares of the different grassland types must be met either at farm or at regional scales (Fig. 4). For each simulation, we estimated the economic value of ecosystem service provision. We then calculated the average ecosystem service value across the 500 simulations for one given share of extensive grasslands (ranging from 0 to 50%). By running 500 simulations for each share of extensive grasslands, we accounted for ecosystem service variability across space. The motivation to use a random distribution (instead of an optimization) is that an optimum allocation of permanent grassland is assumed to be unobservable for the policy maker. This simplification is essential since it allows taking into account the spatial distribution of ecosystem services without a representation of the farmers’ complex land-use decisions. The assumption that the location of the grassland type is a random variable implies that our results with respect to spatial variability must be interpreted as an upper bound for these values. Since the probability that a certain parcel would be management extensively would depend on the farmers’ land-use optimization problem, we would have to expect that the probability would not be fully uniform across the farm or region and thus, the variability would be smaller in reality.

In addition to the variability of ecosystem service provision from the spatial allocation of grassland types, we consider variability in the economic values of the three ecosystem services. For marketable services, we include price variation in our simulations using random draws from a beta distribution based on observed forage and carbon prices. We use willingness to pay estimates for the valuation of non-marketable values of extensively managed grasslands. To reflect the uncertainty of stated preference methods due to underlying methodological differences, we calculated minimum and maximum willingness to pay estimates. The overall economic value results from adding up economic values of forage provision, carbon sequestration and habitat maintenance.

The repeated allocation of the grassland types as well as the price variations and the assumption concerning the willingness to pay levels create variability in the estimation of the economic value of forage provision, carbon sequestration and habitat maintenance from increasing shares of extensive grasslands. We interpret the average estimate as indicator for the economic value of grassland ecosystem services. The standard deviation of $ESV_{total}$ as well as the difference between the maximum and minimum values are used as indicator for potential inefficiencies. To test for the sensitivity of our calculation, we calculated the ecosystem service value also at the scale of agricultural production zones, i.e., regions with the same elevation and similar underlying production conditions. The results are in line with those presented in the main text and our findings remain also on a mid-scale between farm and region (see Supplementary material I).

### Case study and data

**Figure 5:**
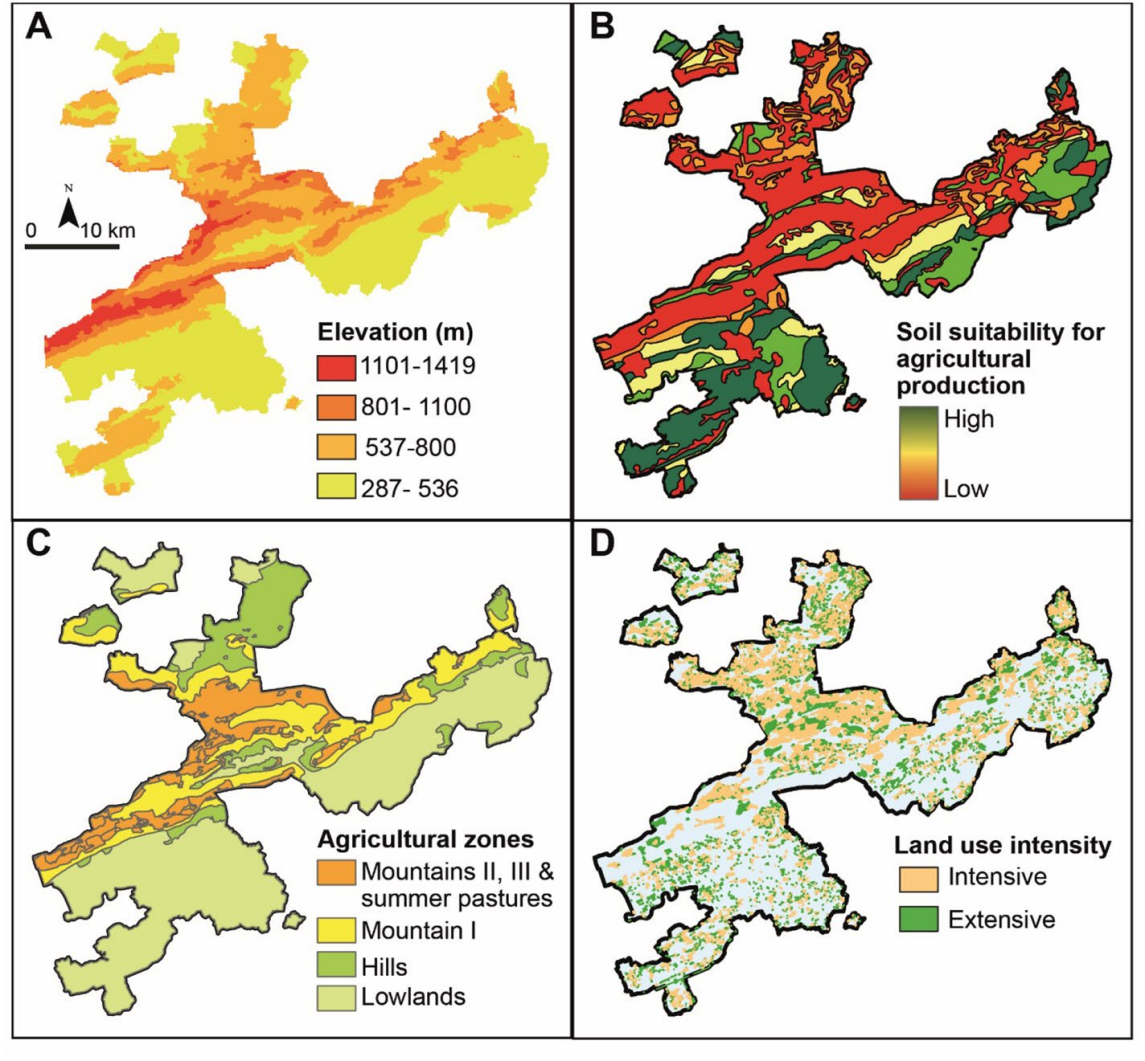
Spatial distribution of the input data for ecosystem service prediction and calculation in the case study region. (**A**) Elevation in m.a.s.l. (**B**) Soil suitability for agricultural production; (**C**) Agricultural production zones. (**D**) Current grassland management in the Canton of Solothurn. Data sources^40,71^ (map produced in ArcGIS 10.7.1 www.esri.com).

We used the Swiss canton of Solothurn as a case study region (Fig. 5). The high share of grasslands in different spatial configurations (i.e., from lowlands to mountains) make the region a suitable case study to analyse the provision of multiple grassland ecosystem services. We focus on permanent grassland, i.e., land used permanently to grow herbaceous forage, and not included in the crop rotation of the farm^40,71^. The case study region can be seen as an exemplary case for diverse farming practices that include a gradient of various landscape archetypes from high intensity livestock farming to low intensity grassland area^72^ Solothurn is located in the northwest of Switzerland and covers an area of 791 km^2^. It presents a wide range of elevations from the lowlands (277 m.a.s.l) to the foothills of the Jura mountains (1445 m.a.s.l). Small-scale and diversified farming systems characterize agricultural land use. Average farm size in the Canton of Solothurn is 26 ha, and average parcel size is 0.9 ha, resulting in a heterogeneous pattern of croplands and grasslands. Predominant agricultural land use is permanent grasslands that covered around 165 km^2^, i.e., 51% of the agricultural area.

In 2017, farmers in the canton of Solothurn cultivated approximately 19% of the total area as extensive grassland. In Switzerland, farmers receive direct payments from the federal government for extensive grassland management (Contribution for Biodiversity, i.e., input-oriented payments), for extensive grassland of highly valuable biodiversity, i.e., output-oriented payments, and for creating networks area between these extensively managed grasslands, i.e., project-oriented payments (which could not be considered in our analysis). On average, these payments in the canton of Solothurn amounted to approximately 600 CHF per ha and year of permanent grassland.

As policy target area $q$, we used (i) the farm scale, i.e., the effective farm size and (ii) the whole canton of Solothurn for the regional scale. In our analysis, we considered all 556 ($n$) milk and meat producing full-time farms from the canton of Solothurn. The mean size of agricultural land is 33 ha per farm. The share of permanent grassland on these farms ranged between 15 and 100%, with a mean share of 51%. On average, farmers cultivated 12% of their land (i.e., 24% of their grassland) as extensive grassland.

In total, we used 19′000 parcels ($i$) in our calculations based on spatial explicit census data for each of the farms provided by the canton of Solothurn. Location characteristics such as elevation, soil quality, slope and landscape diversity were taken from Le Clec'h, et al.^40^. From the original census data, we excluded all parcels which were smaller than 0.1 ha. In addition, we resized very large parcels into smaller units so that all parcels were smaller than 5% of the total farm area and thus can be managed differently. This allowed our random algorithm to re-allocate shares of extensive grassland also to (the border of) larger parcels. Otherwise, farms with only few parcels would not fulfil the restrictions set by the different shares.

For the economic valuation of ecosystem service provision, we used the following data. For forage prices, we used the mean and standard deviation from observed prices in Switzerland over the period of 2010 to 2017 (254-344 CHF per t)^73^. We here assume that the price for forage represent a meaningful proxy for the opportunity costs of a farmer and thus the production value of grassland ecosystem services. Please note that we here consider the price of forage and not the change in revenue of the farmer as a basis for our analysis. With respect to the carbon price, we used two different sources to determine the economic value of carbon sequestration from increasing extensive grassland management. First, we used prices from the CO_2_ European Emission Allowances over the last two years, i.e., 2018 and 2019 (mean price 22.2 CHF; standard deviation 6.2 CHF^74^). These prices, however, do not fully reflect the public cost of internalizing carbon emissions in Switzerland. Thus, we also considered the Swiss CO_2_ levy, i.e., an incentive tax currently only implemented on fossil combustible fuels, to calculate the value of carbon sequestration (96 CHF). The exogenous assumed share of extensively managed grassland $w_{x}$ in our simulation ranged from 0% (baseline scenario) to 50% of grassland. Under current policy regulations, farmers must set aside 7% of extensive grassland as a cross-compliance restriction to be eligible for direct payments in Swiss agricultural policy.

## Supplementary Information

Supplementary Information.
