# Supplementary material for: Economic value of three grassland ecosystem services when managed at the regional and farm scale
Source: Sci Rep. 2022 Mar 9;12:4194. doi: 10.1038/s41598-022-08198-w (PMC8907267; doi:10.1038/s41598-022-08198-w)
Supplement: Supplementary file 1 — Supplementary Information. [file 41598_2022_8198_MOESM1_ESM.pdf]

ECONOMIC VALUE OF THREE GRASSLAND ECOSYSTEM SERVICES WHEN MANAGED AT THE REGIONAL AND FARM SCALE

I. Statistical summary of Results

Economic values for grassland ecosystem services per ha of permanent grassland for three policy target levels i.e., farm, production zone and region.

Level	*Share*	**Minimum**	**5th Percentile**	**1st Quantile**	**Median**	**Mean**	**3rd Quantile**	**95th Percentile**	**Maximum**
**Farm**	*5%*	154	158	162	165	165	168	171	175
	10%	345	351	356	359	359	363	366	372
	15%	427	432	438	441	441	445	449	456
	20%	462	469	475	479	479	484	489	496
	25%	479	484	489	493	494	498	504	508
	30%	478	485	490	495	494	498	504	510
	35%	470	475	480	485	485	489	495	505
	40%	454	457	464	468	468	473	479	486
	45%	431	436	444	447	447	451	457	465
	50%	404	411	417	421	421	424	431	440
**Production zone**	*5%*	374	381	392	400	399	406	416	426
	10%	546	559	570	576	577	584	595	611
	15%	602	624	636	643	643	650	663	686
	20%	639	655	668	674	674	682	691	710
	25%	640	651	664	672	672	680	690	701
	30%	611	642	650	659	659	666	679	691
	35%	597	617	627	635	634	641	652	667
	40%	582	588	600	607	607	615	624	642
	45%	528	553	564	571	572	579	592	607
	50%	497	522	532	537	538	544	555	565
**Region**	*5%*	409	424	432	440	441	448	460	478
	10%	554	574	586	593	593	602	612	622
	15%	639	652	664	671	672	681	692	702
	20%	660	667	680	688	689	696	711	723
	25%	638	651	662	671	671	680	690	716
	30%	645	656	670	678	679	689	701	714
	35%	616	635	647	655	655	663	675	691
	40%	592	603	615	622	623	632	643	656
	45%	550	560	574	583	583	592	604	614
	50%	519	532	542	550	550	558	568	587

II. Data and Code for replication of results

Data and Code are available in the ETH Research Collection: https://www.research-collection.ethz.ch/handle/20.500.11850/386298
